# Identification and In Vitro Antimicrobial Susceptibility of *Brucella* Species Isolated from Human Brucellosis

**DOI:** 10.1155/2014/596245

**Published:** 2014-07-10

**Authors:** Rohaidah Hashim, Norazah Ahmad, Jama'ayah Mohamed Zahidi, B. Y. Tay, Azura Mohd Noor, Sakina Zainal, Hazwani Hamzah, S. H. Hamzah, T. S. Chow, P. S. Wong, K. N. Leong

**Affiliations:** ^1^Bacteriology Unit, Infectious Disease Research Centre, Institute for Medical Research, Jalan Pahang, 50588 Kuala Lumpur, Malaysia; ^2^Microbiology Unit, Department of Pathology, Hospital Pulau Pinang, Jalan Residensi, 10990 Georgetown, Pulau Pinang, Malaysia; ^3^Infectious Disease Unit, Medical Department, Jalan Residensi, 10990 Georgetown, Pulau Pinang, Malaysia

## Abstract

Brucellosis is a world-wide zoonotic disease with a major impact on the public health. Due to the high risk of laboratory acquired infection, limited laboratory investigations were performed on this organism, including detailed identification and susceptibility study. *Brucella melitensis* is the commonest aetiological agent for human brucellosis in this region. The in vitro susceptibility pattern against selected antimicrobial agents was assessed using *E*-test. All isolates were noted to be sensitive to all the antimicrobial agents tested except for rifampicin where elevated MIC > 1 *μ*g/mL was noted in 30 out of 41 isolates tested.

## 1. Introduction

Human brucellosis remains as one of the most common zoonotic diseases being reported worldwide, with approximation of more than 500,000 new cases annually [1]. The disease is caused by* Brucella* spp., which is a fastidious gram-negative bacterium. Disease is acquired upon direct contact with infected animals, consumption of infected dairy products, or inhalation of aerosols generated by any activity during handling of the isolate in the laboratory [2].

The prevalence of brucellosis varies between countries with high endemicity being associated with the Mediterranean countries, Middle East, Southwest Asia, and some areas in Latin America [1, 3]. Implementation of the National Surveillance Programme and a massive vaccination programme of the livestock has resulted in eradication of the disease in Europe, Australia, New Zealand, and Canada [3]. The true incidence and prevalence of Brucellosis in Malaysia are unknown. The incidence of Brucellosis was first reported in Malaysia following the isolation of* Brucella abortus* in 1950 and* Brucella suis* in 1963 among the livestock [4, 5]. Subsequently, cases of human brucellosis surged, urging the initiation of national programme for “The Area-Wise Eradication of Bovine Brucellosis” which was implemented nationwide in 1979. This effort had markedly reduced the prevalence of bovine brucellosis in Malaysia from 3.3% in 1979 to 0.23% in 1988 [5]. Limited adequate Biosafety Level 3 (BSL3) facility required for the handling of Brucella isolates impinged on the proper identification of the organisms. As a consequence, brucellosis cases become underdiagnosed. The sensitivity pattern of the Brucella isolates encountered in this region is also unknown as in vitro susceptibility testing is not done routinely due to above reason. Therefore, clinical cases are treated based on previous literature reports as well as WHO recommendations [6]. Currently, tetracycline, rifampicin, trimethoprim-sulfamethoxazole/cotrimoxazole (SXT), streptomycin, and other aminoglycosides, separately or in combinations, are the most commonly used antimicrobial agents for brucellosis treatment. However, reduced susceptibility of the organisms to streptomycin [7], rifampicin [8, 9], and SXT [10, 11] has been reported recently warranting a proper investigation to assess the susceptibility pattern of the local isolates. The main aim of this study is to ascertain the most common species causing Brucella infection in Malaysia and to determine the susceptibility patterns of these clinical isolates against the commonly used antibiotic agents.

## 2. Materials and Methods 

*Identification Method*. The study included 41* Brucella* spp. isolates which were collected from 2010 to 2011. All isolates were obtained from blood culture except for one, which was derived from tissue biopsy of lumbar area. The isolates come from various parts of Malaysia, mainly Penang 33 isolates (80.6%), Selangor and Kuala Lumpur 2 isolates (4.9%), respectively, and 1 isolate (2.4%) each from Negeri Sembilan, Melaka, Perlis and Kedah. The identification of the organisms was done based on conventional method by taking into consideration the gram stain characteristic, colonial morphology, the fastidious requisite of the organism, and requirement of CO_2_ for growth. The production of urease, oxidase, and hydrogen sulphide, ability to reduce nitrate to nitrite, motility test using semisolid motility medium, and sensitivity to the dyes, basic fuchsin and thionine (at final concentrations of 20–40 *μ*g/mL), were also observed. Agglutination with monospecific antisera for A and M antigens (Animal Health Veterinary Laboratories Agency, UK) is also taken into consideration in identification of the organisms to the genus and species level and to identify the biotype of the organism. This method has been previously described [14, 15].

*Antimicrobial Susceptibility Testing*. The minimal inhibitory concentration (MIC) values of tetracycline (TC), streptomycin (SM), doxycycline (DC), trimethoprim-sulfamethoxazole (SXT), rifampicin (RF), and gentamycin (GM) were determined using the *E*-test (Biomerieux, Sweden) method. Organism inoculum equivalent to 0.5McFarland turbidity was prepared and lawned on Mueller-Hinton agar (BD BBL) supplemented with 5% sheep blood. Each antibiotic strip was individually placed on the inoculated agar and incubated at ambient air, 35°C for 48 hours. Readings were recorded after 48 hours of incubation. The MIC was interpreted as the value at which the inhibition zone intercepted the scale on the *E*-test strip. MIC_50_ and MIC_90_ levels were defined as the lowest concentration of the antibiotic at which 50% and 90% of the isolates were inhibited, respectively. Three Brucella reference strains (*B. abortus* 544,* B. melitensis* 16 M, and* B. suis* 1330) were used as controls for identification, biotyping, and antimicrobial susceptibility testing. In addition to those Brucella reference strains, 2 other organisms,* Escherichia coli* ATCC 25922 and* Staphylococcus aureus* ATCC 29213, were also used as the quality control strain for susceptibility testing.

## 3. Results

All the isolates included in this study were identified as* Brucella melitensis *except for one isolate which was identified as* B. suis. *Serotyping using specific monoclonal antisera revealed that 31 (76%) isolates of* B. melitensi*s were serotype 3, seven (17%) were serotype 2, and two (5%) were from serotype 1.

Interpretation of the MIC value for all antibiotics tested was based on breakpoints for* Brucella spp.* outlined in CLSI [12] except for rifampicin. As no breakpoint for this drug is available, breakpoint for slow-growing bacteria (*Haemophilus influenza*) is referred to [13]. All isolates were sensitive to all antibiotics tested except rifampicin. Only 11 isolates (26%) exhibit MIC ≤ 1 *μ*g/mL for rifampicin. The MIC_50_ and MIC_90_ values of each antibiotic are displayed in Table 1.

Based on the MIC_90_, SXT was noted to be the most potent agent against* Brucella spp.* (0.125 *μ*g/mL), followed by tetracycline and gentamicin (0.19 *μ*g/mL), doxycycline (0.25 *μ*g/mL), streptomycin (0.75 *μ*g/mL), and rifampicin (1 *μ*g/mL). Rifampicin showed the least activity against Brucella, with elevated MIC level of above 1 *μ*g/mL observed in 30 isolates (70%). Therefore, all the drugs were found to be effective to combat brucellosis except for rifampicin. The* Brucella suis* was sensitive to all antibiotics tested.

## 4. Discussion

Brucellosis occurs as sporadic cases with occasional outbreak mainly among people who work with farm animals, especially cattle, sheep, and goats in Malaysia. Cases were also noted among those who consumed raw milk due to traditional belief that it carries some hidden health benefits. The diagnosis of brucellosis is made either by serological detection of the antibody or isolation of the organism. Nonetheless, the gold standard method for the diagnosis of the disease is by isolation of causative agent. As this organism is highly infectious with infective dose of 10–100 bacteria sufficient to cause infection [16], manipulation of these organisms warrants BSL 3 laboratory facilities. Therefore, attempts at isolation and identification of* Brucella* spp. from clinical specimen are not extensively performed. Misidentification of these organisms with other slow-growing gram-negative, nonoxidizer bacteria using commercial test kits, further accentuates the low number of detectable cases. This results in limited data available on the epidemiology of brucellosis in Malaysia. To overcome this devoid of information, we conducted this study, which revealed that human brucellosis in Malaysia is exclusively caused by* B. melitensis*. Our finding is in concordance with the previous reports from different regions of Turkey, Mediteranean and South America Basin [17–19].

There have been many reports on the susceptibility testing for Brucella, based on many different methods [20–22]. Although there was noted to be no significant variation between the methods [23], susceptibility testing using microbroth dilution is the most recommended method for in vitro efficacy testing of antibiotics against* Brucella *sp. However, we opted for *E*-test method for the present study as it was reported to be reliable, reproducible, less labour-intensive, and less time-consuming, as well as imposing less risk of laboratory acquired infection due to lesser manipulation of the organism during the process [17].

The World Health Organization has released recommendations on the use of doxycycline in treating adults with acute infection, combined with either rifampicin or streptomycin for six-week duration [6]. This recommendation is still being used till today. However there were reports that brucellosis with osteoarticular and visceral complications is associated with lesser risk of relapse with triple therapy with streptomycin, rifampicin, and doxycycline [24]. A review and meta-analysis of 30 randomised controlled trials [25] also concluded that preferred treatment should be with combination of doxycycline and gentamicin or triple (e.g., doxycycline with rifampicin and gentamicin) regimens. Even with combination therapy, relapse as high as 10% was still reported [26]. However these relapse cases were associated with inadequate treatment due to improper dosing or poor patient compliance rather than to antimicrobial resistance [8, 27] and therefore antimicrobial susceptibility testing is still not crucial in management of brucellosis cases.

Doxycycline has become the most prescribed tetracycline derivative in the treatment of Brucella infection because of its superior pharmacokinetic nature [28]. In the present study, amongst the tested antimicrobial agents, the MIC breakpoint for both tetracycline and doxycycline was found to be within the susceptible category and among the lowest recorded. This is in concordance with the previous reports [11, 22, 29]. Our MIC_50_ and MIC_90_ values were lower than previously being reported [21, 30]. Therefore this drug is still a potent agent for the treatment of brucellosis. Similar findings were noted with its other derivative agent, tetracycline which displayed even a more potent activity compared to doxycycline. However, the most potent agent with lowest MIC_50_ and MIC_90_ values recorded compared to other agents tested in this study was SXT. This proved that the agent can serve as a good alternative agent for oral treatment. The drug is furthermore cheap, associated with lesser side-effects and is the preferred agent for the treatment of brucellosis among children and pregnant woman [2]. Similar findings which were reported by few other studies proved that the in vitro activity for this drug was consistent [18]. The current MIC value was also much lower than other studies conducted in area endemic of Brucella [22, 30, 31].

Aminoglycosides such as gentamicin, streptomycin, and its derivative were also reported to be effective drugs against brucellosis. A multicentre study reported that combination of gentamicin and doxycycline is considered as of equal efficacy to the recommended regime of doxycycline and streptomycin [32]. Therefore, this may serve as alternative if patients were not tolerating well the first line treatment. The main factor that preludes its wider use is probably the side effect and the parenteral mode of administration. In this study gentamicin showed a good in vitro activity against all the tested Brucella isolates as reported previously in other countries [18, 28, 33]. Similar finding [34] as well as lower [29] and higher [8] MIC values were reported in Turkey for gentamicin.

The breakpoint for rifampicin has not been established, and therefore these organisms cannot be confidently characterised as susceptible, intermediate or resistant. However, based on the breakpoint used, a worrying pattern was observed with our isolates. Elevated MIC level of >1.0 *μ*g/mL were detected among as high as 70% of the isolates. This supports the previous findings of reduced rifampicin susceptibility among Brucella isolates. Our MIC_50_ and MIC_90_ values were similar to some reports [28, 34, 35] however was lower than others [20, 29]. None of the isolates displayed MIC above 2 *μ*g/mL. This elevated pattern is of clinical concern as rifampicin is widely used for the treatment of brucellosis in this country.

## 5. Conclusion

Total eradication of brucella infection has proven to be difficult to achieve as sporadic cases and occasional outbreak were still observed in Malaysia despite the measures previously taken. In this study,* Brucella melitensis* is the main causative agent for human brucellosis in this country. The isolates are susceptible SXT, doxycycline, gentamicin, tetracycline and streptomycin. We are reporting a reduced susceptibility towards rifampicin in majority of our tested Brucella isolates. As routine antimicrobial testing is not feasible in many of the laboratory settings, the use of rifampicin is best avoided as the treatment for brucellosis. A regular screening to monitor for the presence of resistance phenotype if possible, is advisable to ensure early detection of any resistance that may develop and may lead to treatment failure.

## Figures and Tables

**Table 1:** MIC range and MIC_50_ and MIC_90_ values of antimicrobial agents.

Antibiotic	Range (*µ*g/mL)	MIC_50_ (*µ*g/mL)	MIC_90_ (*µ*g/mL)	Breakpoint for susceptibility (*µ*g/mL)
Gentamicin	0.047–0.94	0.125	0.19	≤4^a^
Streptomycin	0.125–1	0.5	0.75	≤8^a^
Cotrimoxazole	0.004–0.25	0.047	0.125	≤2^a^
Doxycycline	0.032–1	0.125	0.25	≤1^a^
Tetracycline	0.023–0.64	0.065	0.19	≤1^a^
Rifampicin	0.38–2	1.5	1	≤1^b^

^a^Clinical and Laboratory Standards Institute (CLSI), 2013. Method for Antimicrobial Dilution and Disk Susceptibility testing of Infrequently Isolated or fastidious Bacteria, Second Edition, M45-A2 [12]. ^b^Clinical and Laboratory Standards Institute (CLSI), 2014. Method for Antimicrobial Dilution and Disk Susceptibility Testing of Haemophilus influenzae, M100-S24 [13].
